# Effects of IQW and IRW on Inflammation and Gut Microbiota in ETEC-Induced Diarrhea

**DOI:** 10.1155/2021/2752265

**Published:** 2021-09-23

**Authors:** Naiyuan Liu, Lingsi Zhou, Jun Fang, Hongmei Jiang, Gang Liu

**Affiliations:** College of Bioscience and Biotechnology, Hunan Agricultural University, Hunan Provincial Engineering Research Center of Applied Microbial Resources Development for Livestock and Poultry, Changsha, Hunan 410125, China

## Abstract

**Methods:**

The mice were randomly distributed into four groups: (a) control (CTRL) group, (b) ETEC group, (c) IQW-ETEC group, and (d) IRW-ETEC group. Villus length and crypt depth were measured after hematoxylin and eosin staining. The inflammatory reaction was analyzed via inflammatory cytokines (i.e., TNF-*α*, IL-1*β*, IL-6, and IL-10) using the enzyme-linked immunosorbent assay (ELISA). The microbiota in the colon was sequenced using 16S ribosomal RNA.

**Results:**

The villus length decreased, the crypt depth decreased, and the expression of inflammatory cytokines (i.e., TNF-*α*, IL-1*β*, IL-6, and IL-10) increased due to ETEC. In the IRW-ETEC and IQW-ETEC groups, the Shannon index decreased (*P* < 0.05). IQW and IRW increased the abundance of *Firmicutes*, *Proteobacteria*, *Clostridiales*, *Lachnospiraceae*, and *Alloprevotella*; contrastingly, it decreased the abundance of *Epsilonproteobacteria*, *Erysipelotrichales*, *Prevotellaceae*, and *Flavobacteriaceae* compared to the ETEC group (*P* <0.05).

**Conclusion:**

This study ascertained that the addition of IQW and IRW could alleviate jejunal inflammation and increase microbiota community diversity.

## 1. Introduction

According to records, diarrhea kills approximately 800,000 children each year [1]. One in ten children worldwide has died due to diarrhea [2]. The cause of diarrhea is complicated; however, pathogenic bacterial infections are one of the major causes. Pathogenic bacteria were detected in 80% of diarrhea patients in Southeast Asia and 72% of diarrhea patients in South America [3].

The traditional treatment of infectious diarrhea involves antibiotics (and sometimes antiviral or antiparasitic medications), which may increase pathogen resistance to drugs. Simultaneously, antibiotics ruin the intestinal microenvironment of the host. Thus, there is much concern about determining alternatives to antibiotics in diarrhea treatment. Natural products, polypeptides, polysaccharides, probiotics, and other substances can be added to the host diet to treat infectious diarrhea; these have received much attention in recent years [4–6]. For example, one study revealed that adding plant polysaccharides to one's diet can alleviate diarrhea symptoms of mice infected with enterotoxigenic *Escherichia coli* (ETEC) [7].

Ile-Gln-Trp (IQW) and Ile-Arg-Trp (IRW) are two active tripeptides extracted from egg whites. Some studies have proved that IQW and IRW have several functions, including lowering blood pressure and cholesterol and antioxidant and anti-inflammatory properties [8–11]. Studies have shown that IQW and IRW can reduce the TNF-induced inflammatory and oxidative stress responses in endothelial cells; these anti-inflammatory and antioxidative effects of IRW and IQW are regulated through the NF-*κ*B signal pathway [12, 13]. It has been shown that IRW can display anti-inflammatory effects by inhibiting p65 protein activity in NF-*κ*B [14]. Additionally, research has shown that IRW can upregulate the expression of nicotinamide phosphoribosyltransferase (NAMPT) protein in mouse muscle cells, improving metabolic levels and alleviating obesity [15]. Our team focused on adding this active peptide to the host diet, exploring the prevention and treatment of intestinal damage. We explored the impact of colitis by adding IRW and IQW to the diet of a DSS-induced colitis mouse model; research showed that IQW could adjust the amino acid levels in serum and regulate intestinal immune function to relieve inflammation; IQW and IRW could reduce oxidative stress induced by DSS by increasing antioxidant enzyme activity; IQW and IRW achieved this by increasing the diversity of the host's intestinal microorganisms and increasing the probiotic biomass [16, 17]. In view of the IQW and IRW effect in the DSS-induced mouse model, we expect IQW and IRW can perform a similar effect in the ETEC-induced mouse model. In this study, we explore the effects of IQW and IRW on jejunal inflammation in an ETEC-induced mouse model.

## 2. Materials and Methods

### 2.1. Experiment Design

The Chinese guidelines for animal welfare were observed for the experimental strategy design. Approval from the Animal Care and Use Committee of Hunan Agricultural University was obtained. In total, 24 male mice (8 weeks, average weight: 23 g) were used in the experiment. All mice were raised at Hunan Agricultural University and fed in a comfortable environment (relative humidity: 53%; average temperature: 24 degrees). In order to simulate natural light conditions, the animals were fed under 12 h of light and 12 h without light. In order to alleviate the stress response caused by the environmental change, mice were given a 3-day adaptation period before the experiment. After 3 days, the mice were randomly divided into four groups: (a) control (CTRL) group (*n* = 6), (b) ETEC group (*n* = 6), (c) IQW-ETEC group (*n* = 6), and (d) IRW-ETEC group (*n* = 6). Groups a and b were given basal diet and natural drinking water for the first 7 days. Group a was given 0.1 mL saline in the first 7 days. Group b was given 0.1 mL 5 × 10^9^ CFU/mL ETEC for 7 days. Mice in groups c and d were put on a basal diet of IQW (93.04% purity, 0.03% mass concentration) and 0.03% IRW (87.91% purity; 0.03% mass concentration), respectively. Meanwhile, 0.1 mL ETEC was given to groups c and d 7 days after the first day of the feeding experiment; this lasted for 7 days. At the end of the 15th day of the feeding experiment, all mice fasted for 12 h and were subsequently weighed before being sacrificed. The acute blood loss method is used to collect blood in mice. The jejunal tissues and colon contents were collected after autopsy; all samples were frozen using liquid nitrogen and stored in a -80°C freezer for further experimentation.

### 2.2. Histological Analysis of the Jejunum

Samples contained different alcohol concentrations (50%, 70%, 80%, 90%, and 95%). For dehydration, a dimethyl benzene ethanol and paraffin (1 : 1) solution was used for the sample embedding processing. The jejunal tissue morphology and tissue damage were analyzed, and the height of intestinal villi and crypt depth were microscope measured using hematoxylin-eosin-stained samples.

### 2.3. Jejunal Tissue Inflammatory Cytokine Detection

Jejunal TNF-*α*, IL-1*β*, IL-6, and IL-10 were detected via enzyme-linked immunosorbent assay (ELISA). Antibodies (anti-TNF-*α*, IL-1*β*, IL-6, and IL-10) were added into the polystyrene HRP-plate well after dilution with carbonate-coated buffer (1 : 100) and placed at 4°C for 12 h overnight. The solution was poured out, and the plates were washed with PBS solution three times. The blocking solution was added to each plate well, incubated at 37°C for 1.5 h. After washing the samples three times using PBS solution, they were incubated at 37°C for 1.5 h. Diluted biotinylated antibodies (goat against mice) were added for 30 min at 37°C. TMB (3,3′,5,5′-Tetramethylbenzidine) substrate was added to each reaction well for the color reaction; the reaction lasted 20 min at 37°C. Sulfuric acid was added to each reaction well for the termination reaction. The absorbance of each reaction was measured at 450 nm.

### 2.4. Microbial Community Analysis

The DNA of colon content samples was extracted, and the purified DNA was used as a template to amplify the variable region of V3+V4 of bacterial 16S rDNA gene by PCR. The PCR products were sent to MicroBio for sequencing analysis. The obtained sequencing results were optimized for OTU-operational taxonomic unit cluster analysis. Alpha diversity analysis (species richness statistics, such as Chao and Ace, and species diversity statistics, such as Shannon and Simpson) was performed using Mothur (version 1.33.3) software. Microbial Ecology (QIIME) was an open-source tool for analyzing the original sequence. PycGootookit6 software was used to deal with the sequence errors and database redundancy in the original data [18]. The optimal overlapping sequence was found by splicing the original sequence [19]. For each sample, the sequence was analyzed using QIIME and the UPARSE application to determine the operational classification unit (OTUs), and the classification data were assigned to each OTUs using the RDP classifier (version 2.2) [20]. The selected sequence of representatives. RDP classifier and Greengenes database were used for classification. Alpha diversity analysis of the jejunal bacterial community was performed using the abundance-based coverage estimator (ACE), bias-corrected Chao richness estimator, Shannon index, and Good's coverage.

### 2.5. Data Analysis

The statistical software package (SPSS V16.0) was used for statistical analysis. The one-way ANOVA method was used to analyze significant differences among groups, and the data were represented as mean ± SD. GraphPad Prism 8 was used to make graphs. *P* values < 0.05 were regarded as significantly different.

## 3. Results

**Figure 1:**
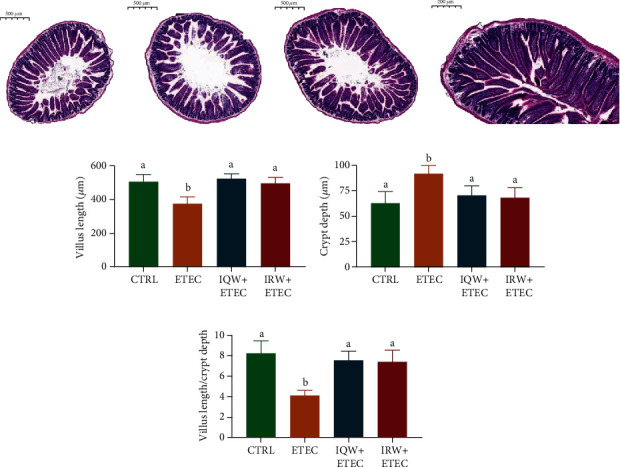
H&E staining results of mouse jejunum (*n* = 6): (a) CTRL, (b) ETEC, (c) IQW+ETEC, and (d) IRW+ETEC. The villus length (e), crypt depth (f), and the ratio of villi length to crypt depth (g); the change in letter denotes a significant difference.

As shown in Figure 1, compared to the CTRL group, the length of intestinal villi in the ETEC group was significantly decreased (*P* < 0.05), and crypt depth was increased considerably (*P* < 0.05), which indicated that ETEC could cause severe jejunal damage. However, compared to the ETEC group, in the IQW-ETEC and IRW-ETEC groups, the length of intestinal villi increased while the crypt depth decreased (*P* < 0.05), which might indicate that IQW and IRW dramatically improve the status of intestinal injury, improving damage recovery.

**Figure 2:**
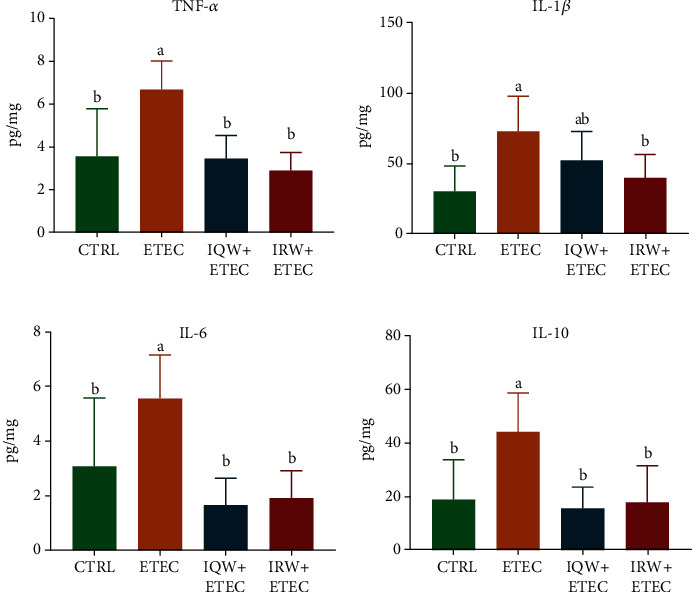
Inflammatory cytokines in the mouse jejunum in each group (*n* = 6): (a) TNF-*α*, (b) IL-1*β*, (c) IL-6, and (d) IL-10; the change in letter denotes a significant difference.

As shown in Figure 2, the contents of inflammatory cytokines (TNF-*α*, IL-1*β*, IL-6, and IL-10) in the ETEC group were significantly higher than those in the CTRL group. The contents of TNF-*α*, IL-1*β*, IL-6, and IL-10 in the IQW-ETEC and IRW-ETEC groups were significantly lower than those in the ETEC group (*P* < 0.05).

**Figure 3:**
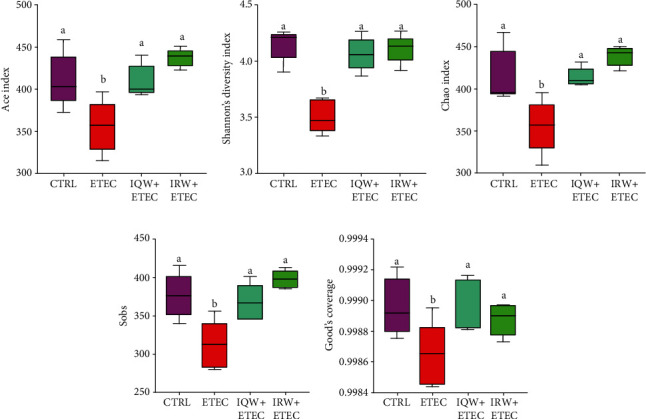
*α*-Diversity of colon microorganisms of mice in each group (*n* = 6), letter (b) denotes a significant difference: (a) Ace index, (b) Shannon index, (c) Chao index, (d) Sobs index, and (e) coverage index.

Sequencing analysis was from the V3-V4 region of 16S rRNA in collected colon content samples.  Figure 3 shows the *α*-diversity analysis of each group. In the ETEC group, the Ace index, Shannon index, Chao index, Sobs index, and Good's coverage were significantly lower than in the CTRL group. Additionally, compared to the ETEC group, the *α*-diversity of the IQW-ETEC and IRW-ETEC groups was significantly increased (*P* < 0.05).

**Figure 4:**
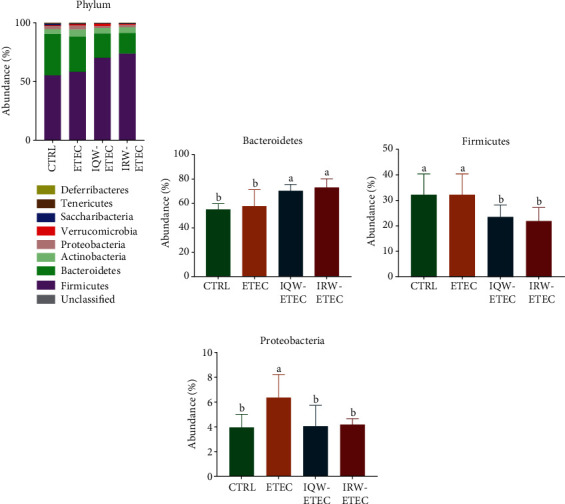
Effect of IQW and IRW treatment on the microorganisms at the phylum level: (a) microbiota of the colon in the four groups at the phylum level (*n* = 6), (b) *Bacteroidetes*, (c) *Firmicutes*, and (d) *Proteobacteria*; the change in letter denotes a significant difference.

Microbiota analysis was conducted on colon content samples; Figure 4(a) shows the relative abundance of collected microorganisms at the phylum level. The abundance of *Bacteroidetes*, *Firmicutes*, and *Proteobacteria* showed advantages in the phylum level. In the CTRL group, the relative abundance of microbiota was *Bacteroidetes* (55.20%), *Firmicutes* (35.33%), and *Proteobacteria* (4.08%). In the IQW-ETEC group, the relative abundance of microbiota was *Bacteroidetes* (58.24%), *Firmicutes* (29.92%), and *Proteobacteria* (6.11%). In the IRW-ETEC group, the relative abundance of microbiota was *Bacteroidetes* (70.21%), *Firmicutes* (20.66%), and *Proteobacteria* (4.77%). In Figure 4(b), IQW and IRW significantly increased the abundance of *Bacteroidetes* compared with the ETEC group (*P* < 0.05). In Figure 4(c), IQW and IRW significantly reduced the abundance of *Firmicutes* in mice compared with the ETEC group (*P* < 0.05). The IRW-ETEC and IQW-ETEC groups were significantly different from the ETEC group (*P* < 0.05), and the proportion of *Firmicutes* in the colon of mice in the IRW-ETEC group also increased to a certain extent. In Figure 4(d), the abundance of *Proteobacteria* in ETEC is significantly higher than in the CTRL group (*P* < 0.05). The abundance of *Proteobacteria* in the IRW-ETEC and IQW-ETEC groups is significantly lower than in the ETEC group (*P* < 0.05).

**Figure 5:**
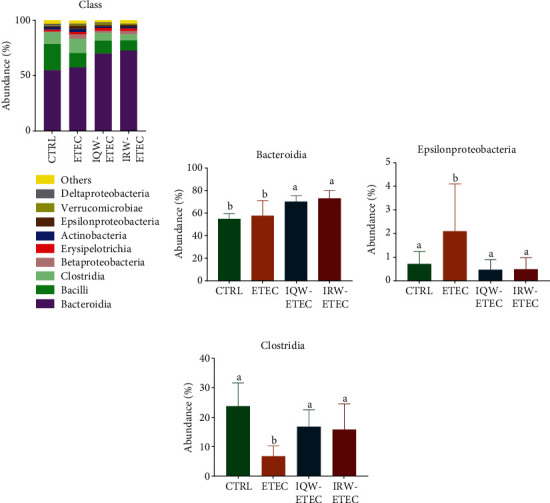
Effect of IQW and IRW treatment on the microorganisms at the class level: (a) microbiota of the colon in the four groups at the class level (*n* = 6), (b) *Bacteroidia*, (c) *Epsilonproteobacteria*, and (d) *Clostridia*; the change in letter denotes a significant difference.

Figure 5(a) shows the nine classes with the most abundant abundance in colon contents. In the CTRL group, *Bacteroides* (54.95%), *Clostridia* (23.7%), and *Bacilli* (10.7%) were the most abundant. In the ETEC group, *Bacteroidetes* (57.5%), *Clostridium* (12.96%), *Bacillus* (12.99%), and *Erysipelotrichia* (3.95%) were the most abundant groups. In the IQW-ETEC group, *Bacteroidetes* (70.1%), *Clostridium* (11.6%), and *Bacillus* (7.1%) were the largest groups. In the IRW-ETEC group, *Bacteroides* (72.9%), *Clostridium* (9.1%), *Bacillus* (5.4%), and *Erysipelas* (3.0%) were the most abundant groups. The results in Figure 5(b) show that both IQW and IRW treatments increased the abundance of *Bacteroidia* in the intestine of mice, and both groups showed significant differences compared with the ETEC group (*P* < 0.05). At the same time, it can be seen from Figure 5(c) that ETEC treatment significantly increased the abundance of *Epsilonproteobacteria* (*P* < 0.05). IQW and IRW treatment could significantly reduce the growth of *Epsilonproteobacteria* (*P* < 0.05). In Figure 5(d), the relative abundance of *Clostridia* in the ETEC group is significantly decreased compared to the CTRL group; IQW and IRW significantly increased the abundance of *Clostridia* compared to the ETEC group.

**Figure 6:**
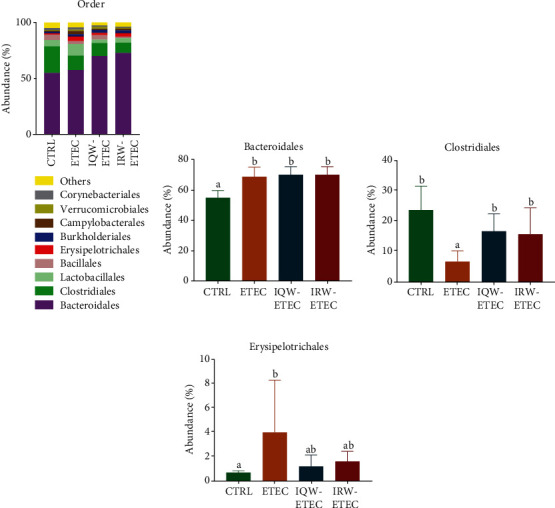
Effect of IQW and IRW treatment on the microorganisms at the order level: (a) microbiota of the colon in the four groups (order level; *n* = 6), (b) *Bacteroidales*, (c) *Clostridiales*, and (d) *Erysipelotrichales*; the change in letter denotes a significant difference.

Figure 6 shows the highest abundance of colon contents at the order level (Figure 6(a)). And in the CTRL group, *Bacteroidales* (54.95%), *Clostridiales* (23.7%), *Lactobacillales* (5.78%), and *Bacillales* (4.9%) were the most abundant groups. In the ETEC group, *Bacteroidetes* (57.53%), *Clostridium* (12.96%), *Lactobacillus* (10.3%), and *Erysipelotrichales* (3.95%) were the most abundant groups. In the IQW-ETEC group, *Bacteroidetes* (70.1%), *Clostridium* (11.6%), *Lactobacillus* (3.1%), and *Bacillus* (4.0%) were the most abundant groups. In the IRW-ETEC group, *Bacteroidetes* (72.9%), *Clostridium* (9.12%), and *Lactobacillus* (3.99%) were the largest groups.  Figure 6(b) shows that ETEC significantly increases the abundance of *Bacteroidales* (*P* < 0.05). The effects of IQW-ETEC and IRW-ETEC were not significant according to the ETEC group (*P* > 0.05).  Figure 6(c) shows the levels of *Clostridiales* in the colon of mice: the group treated with ETEC had significantly decreased amounts compared to the CTRL group (*P* < 0.05); in contrast, the abundance of *Clostridiales* in the IQW-ETEC and IRW-ETEC groups was significantly increased compared to the ETEC group (*P* < 0.05). Thus, the effects of IQW and IRW treatment were effective.  Figure 6(d) shows that ETEC can significantly increase the abundance of *Erysipelotrichales* (*P* < 0.05). The addition of IQW and IRW had an obvious trend in reducing the *Erysipelotrichales* content compared with the ETEC group.

**Figure 7:**
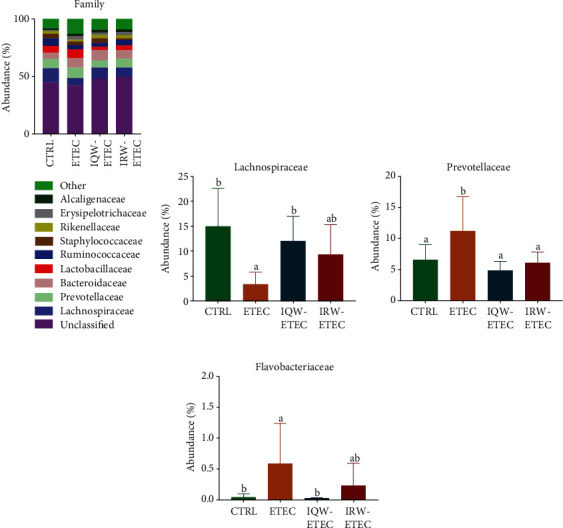
Effect of IQW and IRW treatment on the microorganisms at the family level: (a) microbiota of the colon in the four groups (family level; *n* = 6), (b) content of *Lachnospiraceae*, (c) *Prevotellaceae*, and (d) *Flavobacteriaceae*; the letter change denotes a significant difference.

At the family level, nine families had the highest abundance (Figure 7(a)). In the CTRL group, *Lachnospiraceae* (12.9%), *Prevotellaceae* (8.1%), and *Bacteroidaceae* (5.6%) were the most abundant microorganisms. *Lactobacillaceae* (5.8%) was the most abundant microorganisms in the ETEC group. *Lachnospiraceae* (6.4%), *Prevotellaceae* (9.1%), *Bacteroidaceae* (8.3%), and *Lactobacillaceae* (7.5%) were the most abundant microorganisms in the IQW-ETEC group. The most abundant microorganisms in the IRW-ETEC group included *Lachnospiraceae* (9.6%), *Prevotellaceae* (6.2%), *Bacteroidaceae* (9.2%), and *Staphylococcaceae* (3.9%). *Lachnospiraceae* (7.9%), *Prevotellaceae* (7.6%), *Bacteroidaceae* (7.6%), and *Ruminococcaceae* (4.8%) were the most abundant microorganisms in the IQW-ETEC group. It can be concluded from Figure 7(b) that the *Lachnospiraceae* in the ETEC group is significantly lower than in the CTRL and IQW-ETEC groups (*P* < 0.05). However, there was no significant difference between the ETEC and IRW-ETEC groups. There was a clear trend on increasing the abundance of *Lachnospiraceae*. In Figure 7(c), ETEC significantly increases the abundance of *Prevotellaceae* (*P* < 0.05). And the effect of IQW and IRW was effective (*P* < 0.05). In Figure 7(d), the abundance of *Flavobacteriaceae* is significantly increased by ETEC (*P* < 0.05) compared to the CTRL group. There is a significant decrease of *Flavobacteriaceae* in the IQW-ETEC group compared to the ETEC group. However, there is no significant difference between the ETEC group, IRW-ETEC group, and CTRL group, despite the obvious presence of a trend of *Flavobacteriaceae* decrease in the IRW-ETEC group.

**Figure 8:**
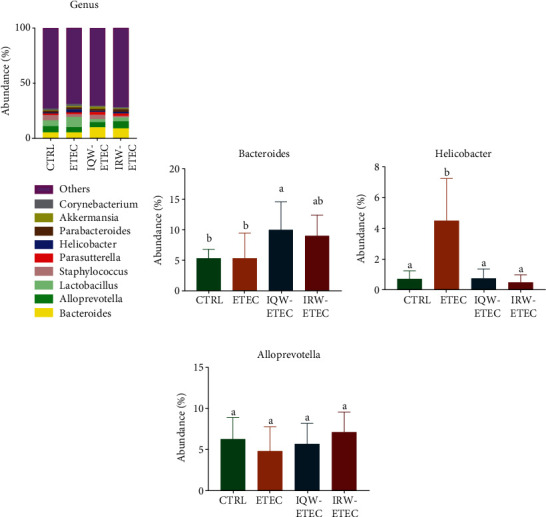
Effect of IQW and IRW treatment on the microorganisms at the genus level: (a) microbiota of the colon in the four groups at the genus level (*n* = 6), (b) *Bacteroides*, (c) *Helicobacter*, and (d) *Alloprevotella*; a letter change denotes a significant difference.

At the genus level, nine genera have the highest abundance (Figure 8(a)). In the CTRL group, *Bacteroides* (5.3%), *Alloprevotella* (5.6%), *Lactobacillus* (5.4%), and *Staphylococcus* (4.7%) were the most abundant groups. In the ETEC group, *Bacteroides* (5.3%), *Prevotella* (4.8%), *Lactobacillus* (9.1%), and *Helicobacter* (2.8%) were the most abundant groups. In the IQW-ETEC group, *Bacteroides* (9.96%), *Prevotella* (4.6%), *Lactobacillus* (2.7%), and *Staphylococcus* (3.8%) were the most abundant groups. In the IRW-ETEC group, *Bacteroides* (8.97%), *Prevotella* (6.4%), and *Lactobacillus* (3.4%) were the largest groups. As shown in Figure 8(b), the abundance of *Bacteroides* in intestinal microorganisms was significantly increased by the IQW compared to the ETEC group (*P* < 0.05), and there was still an obvious trend of *Bacteroides* increase in the IRW-ETEC group compared to the ETEC group.  Figure 8(c) shows that ETEC significantly increased the abundance of *Helicobacter* compared to the CTRL group (*P* < 0.05), and the IQW and IRW significantly increased the abundance of *Helicobacter* compared to the ETEC group (*P* < 0.05). In Figure 8(d), there is a trend of decreasing abundance of *Alloprevotella* after ETEC treatment; there was also a recovery effect after IQW and IRW treatment.

## 4. Discussion

The intestinal tract is one of the most important digestive organs in mammals. Equally important, the intestinal tract also plays an important role in immune function [21]. The intestinal tract is a dynamic and complex system; the intestinal microenvironment is the coexistent result of host and microbiota [22, 23]. The intestinal cells have a strong regenerative capability, with damaged cells recovering within 3 days [24]. The intestinal cells are composed of two types of cell lineages: an absorptive (enterocyte) cell lineage and a secretory (exocrine) cell lineage, both of which originate from intestinal stem cells (ISCs) [25]. Active ISCs are the major actuator for damaged intestinal cells; crypts are the storage region of ISCs [26]. Intestinal villus length and crypt depths are the two common evaluation indexes of intestinal inflammation. Studies have shown that intestinal damage can decrease villus height while increasing crypt depth [27]. Our research highlights the ability of ETEC to cause jejunal damage. Notably, IRW and IQW polypeptides can promote jejunal cell recovery.

After infection, ETEC can adhere to intestinal cells and initiate damage via toxins in a short period [28]. Additionally, ETEC, through the MAPK and NF-*κ*B pathways, causes further inflammatory damage [29]. A previous study pointed out that ETEC infection increased the expression of IL-1*β*, IL-6, TNF-*α*, IL-17, and IL-18 [30]. Our research also determined that ETEC can significantly increase the expression of TNF-*α*, IL-1*β*, IL-6, and IL-10. Moreover, IQW and IRW can significantly alleviate the overexpression of inflammatory cytokines (*P* < 0.05). Accordingly, there is no significant difference between the IQW and IRW groups (*P* > 0.05).

The intestinal microbiota plays an important role in host immunity, digestion, and metabolism and is unique to a specific host body [31]. The mammalian intestinal tract is homeostatically an orderly symbiotic environment; adverse conditions destroy the balance between intestinal microbiota and host [32, 33]. Intestinal inflammation can lead to a disturbance of host intestinal organisms. A study showed that pathogenic bacterial infections, such as *Salmonella enterica* infection, can cause host intestinal inflammation, reducing intestinal microbiota diversity [34]. Our research points out that in the *α*-diversity test of the colon intestinal tissue of experimental mice, the microbial community richness in the ETEC group was significantly decreased compared with that in the CTRL group (*P* < 0.05).  Figure 4 shows that the microbial abundance in the ETEC group was significantly lower than the CTRL, IQW-ETEC, and IRW-ETEC groups (*P* < 0.05). To a certain extent, IQW and IRW can alleviate the decrease in intestinal microbial microorganisms caused by ETEC.

IQW and IRW are two kinds of polypeptides that possess numerous excellent biological activities. A study revealed that IRW functions by regulating and improving the diversity of the intestinal microbiome of the host [35, 36]; our research confirmed this. IRW and IQW can significantly promote intestinal microbiome recovery. In the IQW-ETEC and IRW-ETEC groups, the Shannon index, Sobs index, Chao index, Ace index, and Good's coverage were significantly higher than in the ETEC group.

ETEC reduced the abundance of *Bacteroidetes*. *Bacteroidetes* are the most common intestinal microbes in the human gut, accounting for roughly 50% of the intestinal microbes in a Western person [37]. Some bacteria in the *Bacteroides* genus, such as *Bacteroides fragilis*, have been shown to prevent and treat intestinal diseases. One study showed that *B. fragilis* could alleviate inflammation in the DSS-induced IBD colitis model in mice and alleviate weight loss caused by IBD and inflammation [38]. Some studies have shown that *Bacteroides* have several probiotic effects, such as promoting the digestion of dietary-fiber polysaccharides and the host immunity [39, 40]. Our experimental results showed that IQW and IRW could facilitate the restoration of the host intestinal microbiome environment, improving the abundance of intestinal probiotics in the host intestinal tract and alleviating the jejunal inflammatory response caused by ETEC. A previous study by our research group revealed that IQW could increase the *Bacteroides* biomass, and IRW can increase the abundance of *Clostridium* [41]. And our research showed similar results. Another study pointed out that IRW and IQW intake could increase the abundance of *Firmicutes* and *Actinobacteria* and reduce the proportion of *Bacteroidetes* and *Proteobacteria* [42]. ETEC increased the abundance of *Flavobacteriaceae*, e.g., *F. aquatile* and *F. meningosepticum*, which can cause many diseases, including aquatic diseases (shark fin rot and equine back disease) and neonatal meningitis, respectively [43]. *Flavobacteria* infection is also the infectious agent for chronic skin disease and bacterial gill disease in fish [42]. The experimental results demonstrate that the proportion of *Flavobacteriaceae* in the intestines of IQW-treated mice significantly decreased (*P* < 0.05); after IRW treatment, the proportion of intestinal *Flavobacteriaceae* decreased. Studies have pointed out that IQW and IRW can improve the abundance of *Firmicutes*, *Bacteroidetes*, and *Proteobacteria*, increase the *Lactobacillus* and *Bifidobacterium* biomass, and decrease the abundance of *Helicobacter pylori* and *Verrucomicrobia*[43, 44]. Our research shows similar results, with IRW and IQW improving the abundance of probiotics such as *Firmicutes*, *Proteobacteria*, *Clostridiales*, *Lachnospiraceae*, and *Alloprevotella* and decreasing the abundance of pathogenic bacteria such as *Epsilonproteobacteria*, *Erysipelotrichales*, *Prevotellaceae*, and *Flavobacteriaceae.*

## 5. Conclusions

ETEC can cause jejunal damage, exacerbating the inflammatory reaction. However, IQW and IRW can decrease the expression of inflammatory cytokines, thereby improving the abundance of probiotics such as *Firmicutes*, *Proteobacteria*, *Clostridiales*, *Lachnospiraceae*, and *Alloprevotella* and decreasing the abundance of pathogenic bacteria such as *Epsilonproteobacteria*, *Erysipelotrichales*, *Prevotellaceae*, and *Flavobacteriaceae.*

## Data Availability

The data of this study was available at the corresponding author upon reasonable request.
